# Targeting Selectins Mediated Biological Activities With Multivalent Probes

**DOI:** 10.3389/fchem.2021.773027

**Published:** 2021-12-03

**Authors:** Deepak Ganesh, Prashant Jain, Chethan Devanur Shanthamurthy, Suraj Toraskar, Raghavendra Kikkeri

**Affiliations:** Indian Institute of Science Education and Research, Pune, India

**Keywords:** selectins, cancer, nanoparticles, imaging, drug delivery

## Abstract

Selectins are type-I transmembrane glycoproteins that are ubiquitously expressed on activated platelets, endothelial cells, and leukocytes. They bind to cell surface glycoproteins and extracellular matrix ligands, regulate the rolling of leukocytes in the blood capillaries, and recruit them to inflammatory sites. Hence, they are potential markers for the early detection and inhibition of inflammatory diseases, thrombosis, cardiovascular disorders, and tumor metastasis. Fucosylated and sialylated glycans, such as sialyl Lewisx, its isoform sialyl Lewisa, and heparan sulfate, are primary selectin ligands. Functionalization of these selectin-binding ligands on multivalent probes, such as nanoparticles, liposomes, and polymers, not only inhibits selectin-mediated biological activity but is also involved in direct imaging of the inflammation site. This review briefly summarizes the selectin-mediated various diseases such as thrombosis, cancer and recent progress in the different types of multivalent probes used to target selectins.

## Background

**FIGURE 1:**
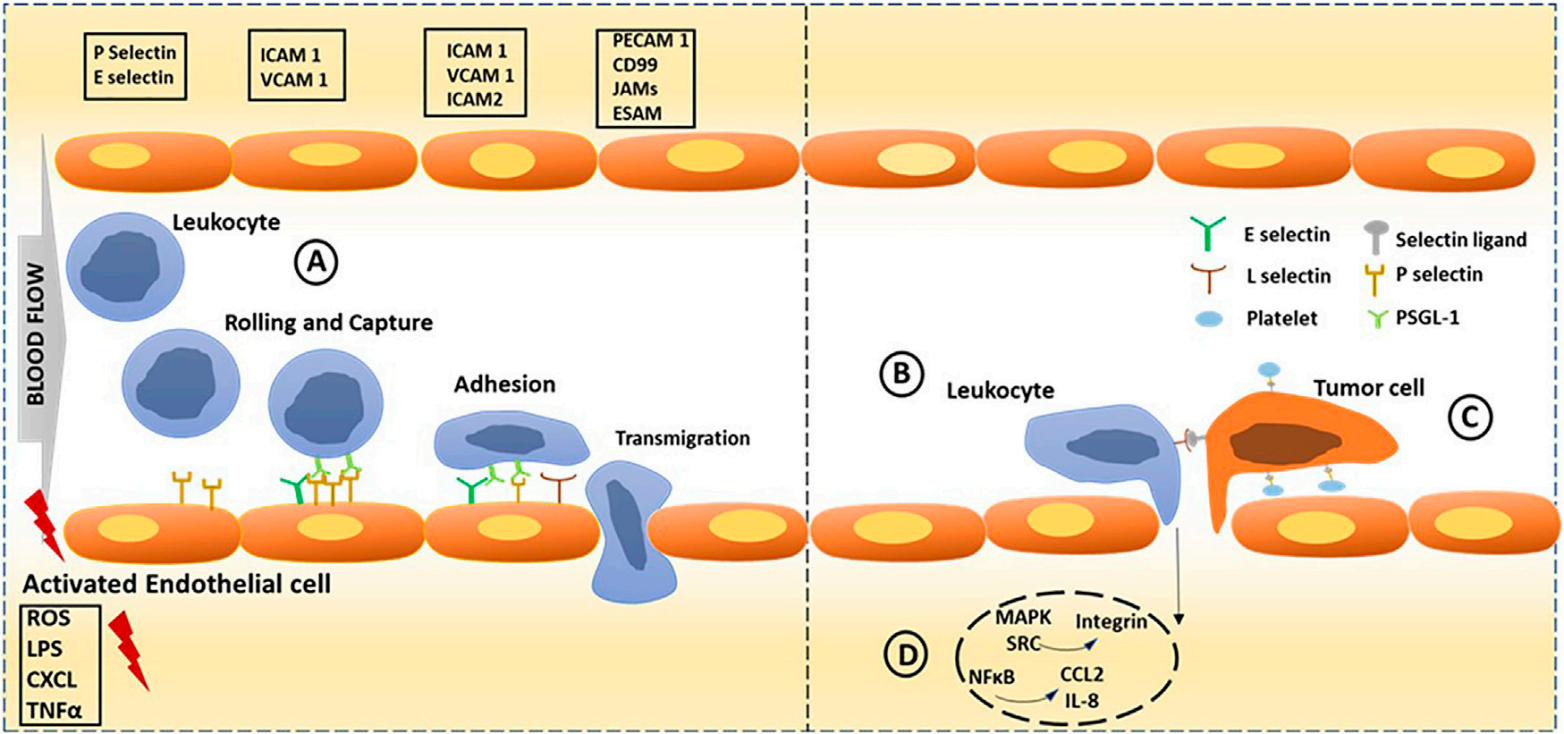
Selectins dependent cell adhesion in normal condition and cancer progression. Endothelial activation is triggered through endogenous and exogenous stimuli from inflamed tissue. **(A)** Leukocyte adhesion cascade could be classified into three steps: rolling; capture, activation and arrest; which is mediated by selectins, chemokines and integrins respectively, progressing to transcellular migration. **(B)** Selectin-triggered endothelial activation leads to leukocyte-assisted tumor cell extravasation. Cytokines produced by the tumor cells lead to endothelial activation and leaky vasculature, resulting in leukocyte recruitment and their extravasation. **(C)** Tumor cell adhesion is promoted by platelets binding to endothelium and tumor cells. **(D)** Intracellular NFκB signalling initiated by selectin binding leading to inflammation through activation of MAPK, SRC pathways leading to activation of integrins and secretion of cytokines like CCL2, IL-8 and TNF-α. Key molecules involved in each step is shown in boxes; PSGL1: P-selectin glycoprotein ligand 1; ICAM 1 and 2: Intercellular adhesion molecule 1 and 2; VCAM 1: Vascular cell adhesion molecule 1; PECAM 1: Platelet/endothelial cell adhesion molecule 1; JAM: junctional adhesion molecule; ESAM: endothelial cell-selective adhesion molecule; ROS: Reactive oxygen species; LPS: lipopolysaccharide; TNFα: tumor necrosis factor-α; CXCL: Chemokine (C-X-C motif) ligand.

The first report of blood cells rolling, adhesion and migration on activated endothelial cell surfaces under shear flow conditions came over a decade ago (Chiara et al., 1999). Later, Giulio et al. reported platelet adhesion on damaged vascular cell walls, resulting in secondary phenomena like blood clotting (De Gaetano et al., 2003). However, the exact mechanism and the molecular level details were unknown until Wagner et al. identified the cell adhesion molecule receptor called selectin on the surface of activated endothelial cells (Wagner et al., 1982). Later, it was quantitatively proved that the selectin family of adhesion molecules plays a significant role in this interaction, particularly in leukocyte recruitment to the site of infection or inflammation and platelet-endothelial cell interaction and aggregation (Pachynski et al., 1998). Leukocytes migrate from capillaries *via* an adhesion cascade which could be classified into three steps: rolling and capture, mediated by selectins, followed by activation and arrest; which is mediated by chemokines and integrins respectively, and arrest, which is mediated by integrins, progressing to transcellular migration. Selectin-triggered endothelial activation leads to leukocyte-assisted tumor cell extravasation and cytokines produced by the tumor cells lead to endothelial activation and leaky vasculature, promoting leukocyte recruitment. Platelets binding to endothelium and tumor cells, promote tumor cell adhesion and this interaction is largely contributed by P-selectin and endothelial activation also triggering leukocyte-assisted tumor cell extravasation. P-Selectin glycoprotein ligand-1 (PSGL-1) and selectin binding triggers intracellular signalling in leukocytes resulting in NFκB, MAPK, and SRC pathways leading to activation of integrins and secretion of cytokines like chemokine (C-C motif) ligand 2 (CCL2), Interleukin-8 and Tumor necrosis factor-α (TNF α) (Figure 1). It has been known since decades that selectins are expressed only at activated platelets and endothelial cells. The activated platelets undergo rearrangement of membrane glycoprotein that in turn is believed to cause rapid series of morphological and biochemical changes. Owing to the overexpression of selectins on activated platelets, covering the entire circulatory system from head to toe, they turned out to be a therapeutic target for several inflammatory diseases, thrombosis, and cardiac diseases (Ludwig et al., 2007). Furthermore, researchers proved that selectins may also promote cancer metastasis and tumor growth (Heinz and Lubor, 2010; McEver and Martin, 1984; Berman et al., 1986; Wu, 1996; Blann and Lip, 1997) (Figure 1), indicating that selectins have important functions besides merely cell adhesion. Therefore, selectin-binding ligand-embedded nanostructures are an exciting tool for designing various biomaterials to target these inflammatory diseases and cardiovascular disorders, as well as cancer and metastasis. Many reviews have already reported the potential applications of selectin targeting in cancer biology and neurobiology. Herein, we specifically focus on multivalent probes such as nanoparticles, polymers and liposomes, and their inherent physical properties, making them an excellent candidate for studying selectin mediated interactions and potential applications.

## Selectins and Its Ligands

Selectins are transmembrane glycoproteins that belong to the C-type lectin family since they exhibit Ca^2+^-dependent glycan binding. The selectin family is made up of P-, E-, and L-selectin with diverse functions and cell adhesion properties. The standard selectin structure contains a C-type lectin domain followed by an epidermal growth factor (EGF)-like domain and short sushi extracellular domains (McEver and Zhu, 2010). P-selectin (CD62P) and E-selectin (CD62E) are expressed on endothelial cells through the activation of pro-inflammatory cytokines, such as tumor necrosis factor (TNF)-α, interleukin -1β (IL-1β), and lipopolysaccharides (Tedder et al., 1995). Generally, E-selectin complements P-selectin binding to recruit leukocytes to the inflammatory site (RP and C, 2010). P-selectin is selectively expressed on activated platelets (Bonfanti et al., 1989). It contributes to platelet adhesion to neutrophils, monocytes, and NK cells, and drives immune cells to injury sites (Chacko et al., 2011). By comparison, L-selectin is expressed on naïve T- and B-cells, myeloid cells, and leukocytes (Rosen, 2004). L-selectin mediates the recirculation of lymphoid cells from lymph nodes to blood circulation and vice versa (Miyasaka et al., 2016). Like the other two selectins, L-selectin also mediates leukocyte adhesion at inflammatory sites (Figure 1). The only difference between the L-selectin adhesion to P and E-selectin adhesion is that it can even occur under high blood flow-rate conditions. These proteins utilize P-selectin glycoprotein ligand-1 (PSGL-1 or CD162) as the primary ligand on leukocytes and hematopoietic stem cells to regulate physiological functions (Moore, 1998). PSGL-1 is a homodimer protein expressed on cell surfaces that undergoes posttranslational modification with sialyl Lewis^x^ (SLe^x^) glycan at *N*-terminals and binds to the selectin carbohydrate-recognition domain (CRD) to regulate the tethering, rolling, and adhesion of leukocytes at the inflammatory site (McEver and Zhu, 2010). In addition to SLe^x^ and P- and L-selectin, E-selectin did not show binding affinity to sulfated forms of SLe^x^ and heparan sulfate/heparin ligands (Brunk and Hammer, 1997). Additionally, several biomimetic analogs of SLe^x^ and HS, such as quinic acid and fucoidan (Amoozgar et al., 2013), have been reported as potential ligands that target selectins. Furthermore, polypeptide sequences such as IELLQAR showed strong inhibition of sialyl Lewis^X^ binding to E-selectin, as endothelial cell-leukocyte interaction and platelet-leukocyte interaction are involved in coagulation, inflammation, and metastasis (Chapon et al., 2009).

## Selectins and Diseases

### Thrombosis and Thrombolysis

Thrombosis is a blood clotting process in veins and arteries, preventing the blood flow in the circulation system and causing illnesses, including acute myocardial infarction, vein thrombosis, and ischemic stroke. Platelets play a fundamental role in preventing blood loss by activating coagulation factors and thereby inducing platelet hemostatic plug. At the injury site, the release of nitric oxide and prostaglandin I_2_ activate endothelial cells, which modulate the expression level of various cell-adhesion molecules, including p-selectin, to regulate platelet adhesion and leukocyte adhesion recruitment, fibrinolysis, inflammation. Hence P-selectin targeting and effective delivery of thrombolytic drug at the endothelial cell/thrombus site could improve the efficacy of thrombolytics (Atherton and Born, 1972). Recently, the vast majority of P-selectin binding ligands scaffolds have been used in thrombolysis and imaging thrombosis.

### Endothelial Dysfunction

Endothelial cells (ECs) play important roles in our lives, as they constitute a thick cell wall layer of arteries, veins, and capillaries. ECs regulate the transport of oxygen, nutrients, and immune cells across different body tissues and maintain blood pressure and blood circulation (Kasteren et al., 2009). Given their diverse biological functions, any inflammation in endothelial cells triggers cell dysfunction, resulting in several diseases. Some diseases, such as stroke, heart disease, chronic kidney failure, angiogenesis, and acute lung injury, are directly associated with vascular function, and other diseases, such as immune disorders, are indirectly associated with dysregulation of immune cell responses at the inflammatory site (Rajendran et al., 2013). Hence, early detection of inflammatory endothelial cells could provide valuable information about several diseases. Previously, it has been shown that E-selectin plays a role in endostatin-mediated anti-angiogenesis (Yu et al., 2004), which promotes and/or inhibits blood vessel formation. Similarly, alveolar-capillary barriers are extensively damaged in acute lung injury (ALI) (Liu, 2019). Thus, abundantly expressed E-selectin on inflammatory endothelial cells acts as a potential therapeutic target for ALI. Furthermore, leukocyte-activated endothelial cell interaction is a hallmark of several neurological disorders, such as multiple sclerosis (MS) (Filippi et al., 2018) and ischemic stroke (Yilmaz and Granger, 2010). Consequently, early diagnosis of this activation is a highly desirable approach.

### Cancer

Cancer cells overexpress selectin binding ligands, as well as selectins on their cell surfaces and promote cancer evasion. These substrates regulate tumor extravasation and support tumor cells rolling on the activated endothelium. Recent immunohistochemistry studies with solid tumor samples revealed that P-selectin expressed on most of the tumor types, including lung (19%), ovarian (68%), lymphoma (78%), and breast (48%) (Shamay et al., 2016). Further, radiation ionization of solid tumor and endothelial cells showed P-selectin translocation on cell membrane (Shamay et al., 2016). Elevated P-selectin has been found to mediate cancer cell adhesion on endothelial cells and platelets, resulting in thrombosis (Merten and Thiagarajan, 2004), cancer mortality (Borsig, 2018), and vasculature (Tedder et al., 1995). Therefore, selective targeting of P-selectins and subsequent anticancer drug delivery could impact different aspects of tumor progression.

## Multivalent Probes

**FIGURE 2:**
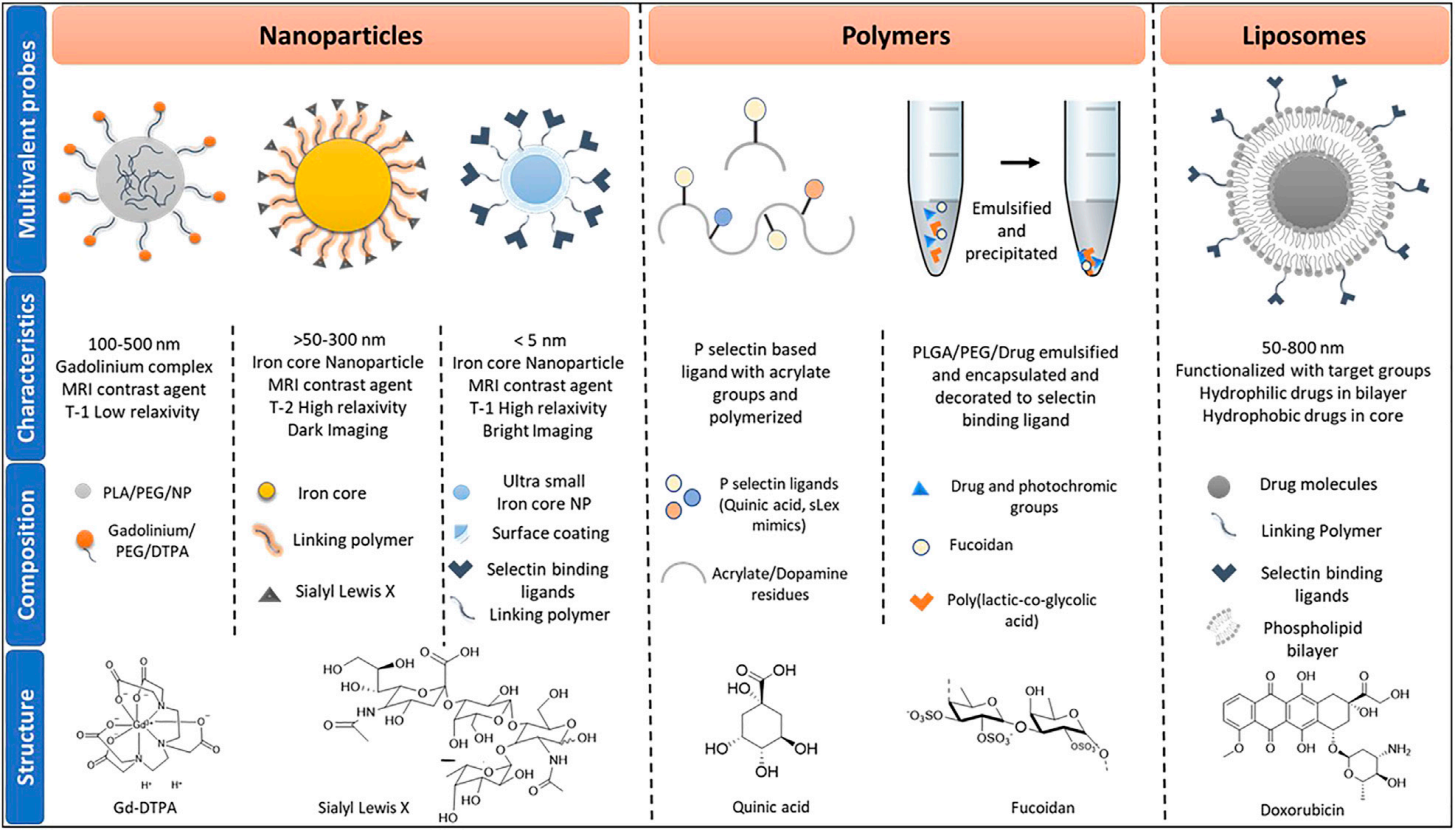
Classes of multiprobes used in imaging and drug delivery, inclusive of their characteristics and composition. Abbreviations used are termed as follows, PLA: Polylactic acid; PEG: Polyethylene glycol; NP: nanoparticles: DTPA: Diethylenetriamine pentaacetate; sLex: Sialyl Lewis x; PLGA: Polylactic-co-glycolic acid.

Multivalent binding governs many biological interactions via the sum of cooperative interactions between ligand-receptors that increase binding avidity rather than affinity (Kiessling et al., 2008). In the last few decades, multivalent probes have shown great promise for gaining new insight into carbohydrate-mediated interactions (Marradi et al., 2013). Consequently, efforts have been made to capture the biological events regulated by selectin-mediated interaction using various multivalent probes. The most prevalent multivalent probes are nanoparticles, liposomes, and polymers (Figure 2).

## Nanoparticles

Usually referred to the particles size of between 1 and 100 nm in diameter. At this tiny size, particles start behaving differently from that of bulk materials. Researchers have exploited the nanoparticles’ inherent optical, electronic, magnetic properties to generate imaging and biomedical applications (Khan et al., 2019). Several types of nanoparticles have been synthesized to target selectin-mediated biological interaction (Table 1). Among them, iron oxide nanoparticles are the most predominant one, due to its inherent magnetic resonance imaging (MRI) contrast agent to develop the real-time imaging of selectin-mediated interactions (Stephen et al., 2011). These magnetic nanoparticles have several advantages over Gadolinium based contrast agent such as Gd-DTPA, which has a low T1 relaxivity (Cheng et al., 2020). The development of iron oxide nanoparticles-based MRI imaging displayed high T2 relaxivity, essential to visualize deep neural networks and tissues. Previously, Ben Davis et al. and Seeberger et al. employed sialyl lewisx conjugated silica iron oxide nanoparticles to study neural inflammation in the ischemic stroke mice model (Farr et al., 2014). MRI imaging of the brain demonstrated the feasibility of imaging endothelial activation *in vivo* after acute stroke (Lu J. et al., 2021). However, the surgical procedure to develop a stroke model enhances P-selectin expression, and therefore the exact level of P-selectin expression is difficult to interpret. Furthermore, the T2 relaxivity of iron oxide nanoparticles that produce dark MRI images induces a high background interface. Hence, IONPs are taken off of the market and looking for an alternative NPs based contrast agent. Recently, ultrasmall iron oxide nanoparticles (USIONPs) of size <4 nm were used as a positive T1 relaxivity MRI contrast agent, as they offer bright MRI imaging and high-resolution, resulting in easy detection of the target (Liu et al., 2019). Recently, Rao et al. reported the synthesis of quinic acid (QA)-coated USIONPs to target P-selectin overexpressed aggressive cancer cells (Narkhede et al., 2019). Working under the same notion, Liu et al. reported the synthesis of USIONPs-PEG-SLex nanoparticles to assess *in vivo* E-selectin expression level in nasopharyngeal carcinoma (NPC) metastasis (Liu et al., 2019). On considering of the inherent magnetic properties of USIONPs, this is a valuable tool for MRI imaging of ischemic stroke.

**TABLE 1:** Class of nanoparticles used in drug delivery and imaging targeted towards selectins.

Target	Ligand	Nanoparticles	Application	References
P-selectin	Fucoidan	Polymer based	Transfer of siRNA	Huang and King (2009)
		Sulfated sialyl lipid Liposomes silica-carbon nano-onion	Drug delivery to activated platelets	Itoh et al. (2014)
		Liposomes	P-glycoprotein inhibitor	Wang et al. (2021)
		Gd based	DOX delivery	Xu et al. (2019)
			Contrast agents/P-selectin inhibitor	Wu et al. (2017), Cheng et al. (2020), Long et al. (2020)
	CD44	Sulfated Hyaluronic acid	Biomedical applications	Bhattacharya et al. (2020)
		PLGA NPs	Multimodal imaging	Li et al. (2021)
	SLe^X^	Ultra-small Iron core NPs	Contrast agents	Chapon et al. (2009), Liu et al. (2019), Narkhede et al. (2019)
E-selectin	Quinic acid	PEG NPs	Paclitaxel delivery	Xu et al. (2018)
	Sialic acid	PEG NPs	DXM delivery	Hu et al. (2017)
	ESTA-MSV	Micelles	Delivery of microRNA	Ma et al. (2016)
L-selectin	Antibody	Gadolinium complex	Biosensors	Wang et al. (2010)
	Sialic acid	Silver NPs	Bacteria mediated therapy	Mi et al. (2021)
P/E-selectin	SLe^X^	Silica Iron oxide	Imaging early inflammation and stroke	Farr et al. (2014)

Abbreviations: DOX, Doxorubicin; DXM, Dexamethasone; ESTA-MSV, E-Selectin targeted multistage vector; Gd(III), Gadolinium; PLGA, Poly Lactic-co-Glycolic acid; PEG, Polyethylene glycol; SLe^X^, Sialyl-Lewis^X^.

Bacterial mediated tumor therapy (BMTT) is an attractive target to cure cancer (Liu, 2019). However, neutrophil recruitment to the tumor site eliminates the bacteria and induces an immunological barrier for BMTT (Mi et al., 2021). As a result, neutrophil-depleting strategies have been employed to improve BMTT cancer therapy. Recently, Rong and co-worked synthesized sialic acid-coated silver nanoparticles to target L-selectin on neutrophils (Mi et al., 2021). These glyco-AgNPs showed neutrophilic depletion and increased salmonella bacteria’s efficacy, which directly killed tumor cells and demonstrated superior therapeutic potentials (Mi et al., 2021). However, the pathogenicity of the bacteria can also have a large effect real-time application of BMTT.

## Polymers

Polymers are powerful multivalent probes, as they offer a wide range of molecular weight, cheaper, biocompatible and readily scalable materials. There are two types of polymeric systems readily used in selectin-mediated studies. In the first set of selectin-targeted polymers, selectins specific ligands, such as quinic acid, sLex mimics, were functionalized on acrylate or dopamine residue and employed polymerization strategy to obtain desired polymeric nanoparticles (Amoozgar et al., 2013). However, the functional groups such as carboxylic acid and hydroxy groups quench the free radical polymerization, resulting in low efficacy and high polydispersity. Alternatively, polymeric nanoparticles were formulated by the emulsion-solvent evaporation method encapsulated with drug molecules and selectin binding ligands (Kona et al., 2012). This method offers several advantages including the mild condition to formulate biocompatible and biodegradable nanoparticles without compromising the activity of drug. Moreover, the nanoparticles prepared by this method offer control delivery of drug molecules. Nguyen and co-workers reported the synthesis of drug (dexamethasone) and fluorescent tag (6-coumarin dye) and glycoprotein Ib (GPIb; p-selectin binding ligand)-loaded poly(lactide-co-glycolide) nanoparticles to target P-selectin overexpressed endothelial cells and drug delivery (Inyang et al., 2020). Similarly, Ran and co-workers synthesized polymeric nanoparticles composed of photothermal agents such as nanocarbons, doxorubicin and perfluropentane encapsulated polymeric nanoparticles coated with platelet membrane (Li et al., 2021). These multi-component nanoplatelets were used to demonstrate breast cancer theranostic. Mizrachi et al. synthesized fucoidan-based fluorescent nanoparticles loaded with BYL719, a PI3Kα inhibitor to target squamous cell carcinoma (Mizrachi et al., 2017). Lee et al. synthesized fucoidan-doxorubicin nanoparticles to target P-selectin overexpressed cancer cells. While, Chuang et al. synthesized fucoidan-coated polypyrrole nanoparticles to target cancer cells and generate photothermal treatment of tumors (Lu K.-Y. et al., 2021). Overall, these results all show the huge potential of polymer-based nanoparticles in real-time applications of cancer therapy.

## Liposomes

Liposomes are one of the attractive nanocarriers for controlled cargo delivery, composed of lipid bilayers in a discrete aqueous environment. They can host both hydrophilic drug molecules in the aqueous centre and hydrophobic molecules between the lipid bilayers and display a large surface area to functionalize biological ligands, including carbohydrates, peptides, and proteins (Sercombe et al., 2015). Liposomes are extensively used to design selectin-mediated drug delivery and imaging system to target various diseases (Guimarães et al., 2021). Li et al. (2020) synthesized sialic acid-modified doxorubicin based liposomes to target and kill peripheral blood neutrophils *via* sialic acid-L-selectin interaction to reduce the accumulation of neutrophile at the rheumatoid arthritis (RA) disease site. Similarly, Matsumura et al. synthesized sialyl lewis X-modified doxo-liposomes to target injured vessel walls to prevent stenosis after angioplasty (Tsuruta et al., 2009). While, Zalipsky et al. reported silyl lewis X liposomes to develop antiadhesion molecules (DeFrees et al., 1996). Azab et al., prepared bone marrow microenvironment destructing inhibitor modified P-selectin glycoprotein ligand-1 conjugated liposome to target multiple myeloma-associated endothelium (Azab et al., 2012). However, single-ligands often fail to target the dynamic microenvironment of the tumor, particularly metastasis cancer cells. To improve the accuracy in targeting metastasis cancer cells, multi-ligand embedded liposomal nanoparticles have been synthesized. Here, P-selectin-specific ligands, integrin-targeting peptides, fibronectin targeting peptides, and epidermal growth factor receptor (EGFR) targeting peptides were assembled on a single liposome to target more than one receptor overexpressed on cancer cells (Peiris et al., 2018). By employing such multi-ligand strategies, highly sensitive and precise imaging of early-stage cancer cells metastasis was achieved (Doolittle et al., 2015; Peiris et al., 2018). Overall, selectin-ligands conjugated liposomes have shown enormous potential in theranostics. However, the poor cost-effectiveness of liposome-based drug-delivery limited its clinical translation.

## Future Direction and Summary

This mini-review highlights the impressive work carried out by different research groups functionalizing the nanoprobes with selectins targeting ligands and their potential theranostic applications. Nanoprobes’ distinct magnetic, fluorescent, and drug carrier ability was used to probe selective targeting and drug delivery to treat cancer, inflammatory diseases, and thrombosis. However, the main challenge is the weak and reversible binding affinity of native ligands such as SLe^x^ and SLe^a^ to selectins (Brunk and Hammer, 1997). Hence, there is a lot of scope to design and identify synthetic ligands from heparan sulfate to improve selectins’ binding affinity and specificity. Furthermore, selectin nanoprobes targeting tumor microenvironment and brain strokes must pass through specific barriers, including blood brain barrier (BBB) and extracellular matrix/stromal cell barriers. These barriers drastically influence the number of nanoparticles delivered to the active sites. Hence there is a great deal of interest in modifying nanoprobes to improve their efficacy.

In summary, leukocyte-activated endothelial and platelet-endothelial cell interaction plays a critical role in various human disorders, including thrombosis, stroke, heart disease, kidney failure, tumor growth metastasis. Hence selectin-binding ligand conjugated nanoprobes serve as a potential marker for the early detection of these diseases and their prevention and more effective treatment. SLe^X^, fucoidan, quinic acid and selectin-binding peptides have been used for quantitative targeting selectin-mediated interactions. In addition, SLe^X^-Iron nanoparticles have been extensively exploited for MRI imaging of brain stroke and cancer metastasis.
